# A Case of Eosinophilic Esophagitis Accompanying Familial Mediterranean Fever

**DOI:** 10.1155/2017/6863921

**Published:** 2017-01-31

**Authors:** Pejman Rohani, Mehri Najafi Sani, Mitra Ahmadi, Vahid Ziaee

**Affiliations:** ^1^Children's Medical Center, Pediatrics Center of Excellence, Tehran University of Medical Sciences, Tehran, Iran; ^2^Department of Pediatrics, Shahid Beheshti University of Medical Sciences, Tehran, Iran; ^3^Department of Pediatrics, Tehran University of Medical Sciences, Tehran, Iran; ^4^Rheumatology Research Center, Tehran University of Medical Sciences, Tehran, Iran

## Abstract

*Background*. Eosinophilic esophagitis is an inflammatory condition where there is a dense infiltration of eosinophils typically exceeding fifteen cells per high power field. Familial Mediterranean fever is an autosomal recessive disorder characterized by brief, acute, and self-limited episodes of fever and polyserositis that recur at irregular intervals.* Case Presentation*. A three-year-and-nine-month-old Iranian girl was admitted to our center. The patient's parents complained of a history of abdominal pain, poor appetite, and poor weight gain from 1.5 years ago and episodes of food impaction after starting solid foods. Eosinophilic esophagitis was diagnosed based on histology. Because of continuing abdominal pain after treatment of eosinophilic esophagitis, the episodic nature of disease, and the presence of fever with pain, screening for familial Mediterranean fever mutation was performed and the patient was found to be heterozygote for Mediterranean fever.* Conclusion*. We have reported a case of eosinophilic esophagitis coexisting with familial Mediterranean fever which has not been described previously.

## 1. Introduction

Eosinophilic esophagitis (EOE) is an inflammatory condition in which there is a dense infiltration of eosinophils more than 15 per high power field (hpf) [1–3]. Presenting symptoms include vomiting, feeding problems, chest or epigastric pain, and dysphagia with occasional food impaction [1].

Clinical definition is provided by no response of gastroesophageal reflux symptoms to high dose of proton pump inhibitors, a normal PH study, and the spectrum of clinical symptoms characterizing its presentation and differentiating it from other esophageal diseases [4]. Definite diagnosis is based on histology by finding more than 20–40 eosinophils per hpf [5].

Familial Mediterranean fever (FMF) is an inherited disorder characterized by recurrent, irregular, and self-limited attacks of chest and abdominal pain associated with fever [6, 7]. The gene responsible for FMF is mapped on the short arm of 16p 13.3. It is also designated MEFV (ME for Mediterranean and FV for fever). Approximately 70% of patients with clinical manifestations of FMF are heterozygous and the most common missense mutation is M6947 [1]. Genetic testing for the FMF gen confirms the diagnosis of FMF [1].

## 2. Case Presentation

A three-year-and-nine-month-old female patient was referred to our hospital with frequent vomiting, abdominal pain, and episodes of food impaction from 1.5 years ago. She was the first child of healthy nonrelative parents. Her parents did not have allergic history and her delivery was at the age of 35 weeks because of preterm labor. Birth body weight was 2000 g, birth height was 46 cm, and head circumference at birth was 34 cm.

She had a history of poor appetite and poor weight gain from infancy. At the age of 3 years when she was first brought for evaluation, her physical examination was as below:

Body weight was 12 kg (between 5 to 25 percentiles) and head circumference was 95 cm (50−75 percentiles).

*Laboratory Findings Revealed*. Lab findings were as follows: WBC count: 7280; Hb = 12.2; platelets: 201000; MCV = 79.4 (other indices of CBC were at normal range for age). Biochemistry, liver function, urine analysis/culture, and sediment tests were all normal.

Her abdominal ultrasound did not show any abnormal finding. Evaluation of frequent vomiting and food impaction was performed by upper GI series which showed mild prominence of gastric mucosal pattern and stoppage of barium at pyloric canal. Small bowel follow through and upper endoscopy were both normal. Macroscopic appearance of esophagus, stomach, and duodenum was normal but histological appearance revealed 50–70 eosinophils per hpf of esophagus (EOE).

Stomach and duodenum did not have special pathologic changes. She was given the treatment for EOE, namely, elimination of some foods based on the skin prick test (elimination of milk and dairy products) and proton pump inhibitors administration (PPI).

After the start of treatment significant improvement was observed in appetite, food refusal, and food impaction, but she still had episodes of abdominal pain which was accompanied with fever lasting 3 days every 3-4 months. When we asked parents about a history of fever they indicated a history of low grade fever less than 38.5 Celsius most of the time, detected by parents before diagnosis of EOE, but they believed it was related to viral infection according to previous general physician examinations. They also mentioned rare cases of fever above 39.5 Celsius. There were no other significant findings in history and exam as the family history was negative for inflammatory bowel disease (IBD) or any other inflammatory diseases and no oral ulcer or arthropathy was found in exam.

Subsequently the patient was evaluated for infective diseases and then inflammatory diseases including SLE, IBD, and hyper-IgD syndrome but all studies were negative. At last she was screened for FMF mutation which showed heterozygote mutation at codon V7216A.

Colchicine was started for the patient and clinical response was observed regarding her abdominal pain and periodic fever. At the end of two months follow-up her endoscopy revealed 2-3 eosinophils per hpf of specimen, her gastrointestinal complaints subsided, and her weight gain was improved.

## 3. Discussion

EOE is an inflammatory disease which is characterized by dense infiltration of eosinophils, vomiting, feeding problems, chest and epigastric pain, and dysphagia [8]. Clinical definition is provided by the absence of responsiveness to high dose of PPIs [4]. Definite diagnosis is based on histology [5].

FMF is characterized by brief but severe self-limiting attacks of abdominal pain and peritonitis, sinusitis, and pleurisy. In the past, diagnosis of FMF was based on clinical manifestations, response to colchicine, and exclusion of other diseases with similar presentation. Nowadays besides clinical symptoms, confirmation of FMF is by mutation analysis [9, 10].

Our patient had EOE evaluation for vomiting, food impaction, and abdominal pain. Because of the continuation of abdominal pain after treatment for EOE and the periodic nature of the disease and fever, FMF was suspected and confirmed by mutation analysis. There are several reports of FMF accompanying other disorders including polyarteritis nodosa, hypereosinophilia, and eosinophilic gastroenteritis [11–13], but there is not enough data to include hypereosinophilia among the diseases that accompany FMF. It should be noted that since the present report is the first report of FMF and EOE being diagnosed in the same patient it might be a coincidental finding. Also FMF and Henoch-Schönlein Purpura (HSP) have been previously reported in patients as coincidental diseases [14, 15]. Today researchers believe some types of vasculitis are more common in FMF and maybe HSP, as the most common vasculitis in children, is a risk factor for recurrent or persistent FMF [16]. To our knowledge this case is the first presentation of FMF and EOE, so this combination should be considered in patients with FMF and unexplained gastroenterologic symptoms of EOE and vice versa. Future reports of this combination of diseases are needed before association between these two diseases could be confirmed.

## 4. Conclusion

We have reported a case of eosinophilic esophagitis coexisting with familial Mediterranean fever which has not been described previously.

## References

[1] Nguyen N., Furuta G. T., Menard-Katcher C. (2015). Recognition and assessment of eosinophilic esophagitis: the development of new clinical outcome metrics. *Gastroenterology and Hepatology*.

[2] Chawla N., Deshmukh M., Sharma A., Patole S. (2016). Strategies for medical management of pediatric eosinophilic esophagitis—a systematic review. *Journal of Pediatric Gastroenterology and Nutrition*.

[3] Liacouras C. A., Wenner W. J., Brown K., Ruchelli E. (1998). Primary eosinophilic esophagitis in children: successful treatment with oral corticosteroids. *Journal of Pediatric Gastroenterology and Nutrition*.

[4] Noel R. J., Putnam P. E., Rothenberg M. E. (2004). Eosinophilic esophagitis. *New England Journal of Medicine*.

[5] Fox V. L., Nurko S., Teitelbaum J. E., Badizadegan K., Furuta G. T. (2003). High-resolution EUS in children with eosinophilic “allergic” esophagitis. *Gastrointestinal Endoscopy*.

[6] Danovitch G. M., Le Roith D., Sobel R. (1979). Amyloid goiter in familial Mediterranean fever. *Clinical Endocrinology*.

[7] Levy M., Yaffe C. (1978). Testicular function in patients with familial Mediterranean fever on long-term colchicine treatment. *Fertility and Sterility*.

[8] Faubion W. A., Perrault J., Burgart L. J., Zein N. N., Clawson M., Freese D. K. (1998). Treatment of eosinophilic esophagitis with inhaled corticosteroids. *Journal of Pediatric Gastroenterology and Nutrition*.

[9] Sohar E., Gafni J., Pras M., Heller H. (1967). Familial Mediterranean fever. A survey of 470 cases and review of the literature. *The American Journal of Medicine*.

[10] Ahmadinejad Z., Mansori S., Ziaee V. (2014). Periodic fever: a review on clinical, management and guideline for Iranian patients—Part I. *Iranian Journal of Pediatrics*.

[11] Basaranoglu M., Mert A., Tabak F., Apaydin S., Aktuglu Y., Ozdogan H. (1999). A case of familial Mediterranean fever and polyarteritis nodosa complicated by spontaneous bilateral perirenal and subcapsular splenic haemorrhage. *Rheumatology*.

[12] Keklik M., Unal A., Sivgin S. (2014). The coincidence of familial mediterranean fever and hypereosinophilia in a patient with hereditary elliptocytosis. *Indian Journal of Hematology and Blood Transfusion*.

[13] Kocak G., Kocak E., Yilmaz Ş. R. (2010). MEFV gene mutations in a patient with eosinophilic gastroenteritis. *Southern Medical Journal*.

[14] Ertan P., Tekin G., Şahin G. E. (2011). A case of henoch-schönlein purpura with P369S mutation in MEFV gene. *Iranian Journal of Pediatrics*.

[15] Ben-Chetrit E., Yazici H. (2016). Non-thrombocytopenic purpura in familial Mediterranean fever-comorbidity with Henoch-Schönlein purpura or an additional rare manifestation of familial Mediterranean fever?. *Rheumatology (Oxford)*.

[16] Aksu K., Keser G. (2011). Coexistence of vasculitides with familial Mediterranean fever. *Rheumatology International*.

